# Extrinsic factors regulate partial agonist efficacy of strychnine-sensitive glycine receptors

**DOI:** 10.1186/1471-2210-4-16

**Published:** 2004-08-09

**Authors:** Jeffrey S Farroni, Brian A McCool

**Affiliations:** 1 Department of Medical Pharmacology & Toxicology, Texas A&M University System Health Science Center, College Station TX 77843, USA; 2 Department of Marine Biology, Texas A&M University at Galveston, Galveston TX 77553, USA; 3 Department of Physiology & Pharmacology, Wake Forest University School of Medicine, Winston-Salem NC 27157, USA

## Abstract

**Background:**

Strychnine-sensitive glycine receptors in many adult forebrain regions consist of alpha_2 _+ beta heteromeric channels. This subunit composition is distinct from the alpha_1 _+ beta channels found throughout the adult spinal cord. Unfortunately, the pharmacology of forebrain alpha_2_beta receptors are poorly defined compared to 'neonatal' alpha_2 _homomeric channels or 'spinal' alpha_1_beta heteromers. In addition, the pharmacologic properties of native alpha_2_beta glycine receptors have been generally distinct from receptors produced by heterologous expression. To identify subtype-specific pharmacologic tools for the forebrain alpha_2_beta receptors, it is important to identify a heterologous expression system that closely resembles these native glycine-gated chloride channels.

**Results:**

While exploring pharmacological properties of alpha_2_beta glycine receptors compared to alpha_2_-homomers, we found that distinct heterologous expression systems appeared to differentially influence partial agonist pharmacology. The β-amino acid taurine possessed 30–50% efficacy for alpha_2_-containing receptor isoforms when expressed in HEK 293 cells. However, taurine efficacy was dramatically reduced in L-cell fibroblasts. Similar results were obtained for β-alanine. The efficacy of these partial agonists was also strongly reduced by the beta subunit. There were no significant differences in apparent strychnine affinity values calculated from concentration-response data between expression systems or subunit combinations. Nor did relative levels of expression correlate with partial agonist efficacy when compared within or between several different expression systems. Finally, disruption of the tubulin cytoskeleton reduced the efficacy of partial agonists in a subunit-dependent, but system-independent, fashion.

**Conclusions:**

Our results suggest that different heterologous expression systems can dramatically influence the agonist pharmacology of strychnine-sensitive glycine receptors. In the systems examine here, these effects are independent of both absolute expression level and any system-related alterations in the agonist binding site. We conclude that complex interactions between receptor composition and extrinsic factors may play a significant role in determining strychnine-sensitive glycine receptor partial agonist pharmacology.

## Background

It has been well established that the amygdala is important in the acquisition and maintenance of fear/anxiety-related behaviors [1]. Strychnine-sensitive glycine receptors have recently been found in the adult rat basolateral amygdala (BLA) using whole cell and intracellular electrophysiology [2,3]. Reverse transcription polymerase chain reaction on whole BLA tissue and single cells revealed a prominent expression of α_2 _mRNA; and these receptors are likely to be α_2_β heteromers due to their low picrotoxin sensitivity [4]. This finding is consistent with prominent BLA 'general' immunoreactivity for α/β subunit protein but no apparent α_1_-specific protein expression [3]. A similar enrichment of α_2_/β heteromers is also evident in striatal cholinergic interneurons [5]. It is quite possible then that the α_2_β strychnine-sensitive glycine receptors present in the adult BLA and other forebrain areas represents a receptor population that could be functionally distinguished from those found in the spinal cord. Because the BLA regulates a number of anxiety- or fear-related behaviors [6], it is possible that this population of strychnine-sensitive glycine receptors may represent a novel therapeutic target for anxiety disorders. To insure that novel α_2_β compounds possess an appropriate therapeutic index, the pharmacology of these forebrain glycine receptors must be elucidated and extensively compared with the spinal isoform.

There have been conflicting reports regarding the details of glycine receptor pharmacology when expressed in heterologous systems. For example, taurine acts as a partial agonists (ca. 50% efficacy compared to glycine) for GlyRα_1 _expressed in *Xenopus ooctyes *[7] whereas it shows nearly full agonist efficacy for GlyRα_1 _expressed in HEK 293 cells [8]. Compared to GlyRα_1_, taurine efficacy is even weaker for GlyRα_2 _(ca. 5–10% efficacy) when expressed in *Xenopus *oocytes [7]. However, native GlyRα_2_β receptors expressed by BLA neurons possess >50% efficacy for taurine and almost full efficacy for β-alanine [2]. While these results might initially be dismissed as expression system-dependent phenomena, brain region-specific effects are also evident in the literature. Taurine has markedly different efficacies at glycine receptors expressed by isolated adult lateral/basolateral amygdala neurons [2], adult hypothalamic magnocellular neurons [9], and juvenile spinal cord neurons [10]. It is therefore possible that the mechanisms regulating brain region-specific effects are related to those governing the divergence among heterologous expression systems. However, such mechanisms have not been systematically investigated, despite their potential usefulness in understanding region-to-region pharmacologic heterogeneity evident for some native receptors.

This study utilizes whole-cell patch clamp electrophysiology to examine the influence of distinct heterologous expression systems on the β-amino acid pharmacology of glycine receptors composed of distinct subunit combinations. We have focused on the α_2 _and α_2_β receptors since these appear to be the predominate isoforms found in the embryonic and adult forebrain, respectively. Our results provide potentially important insight into the types of mechanisms that may govern brain region-to-brain region variation in glycine receptor pharmacology. Several aspects of this work have appeared in abstract form [11,12].

## Results

### Subunit- and system-dependent effects on glycine pharmacology

**Figure 1:**
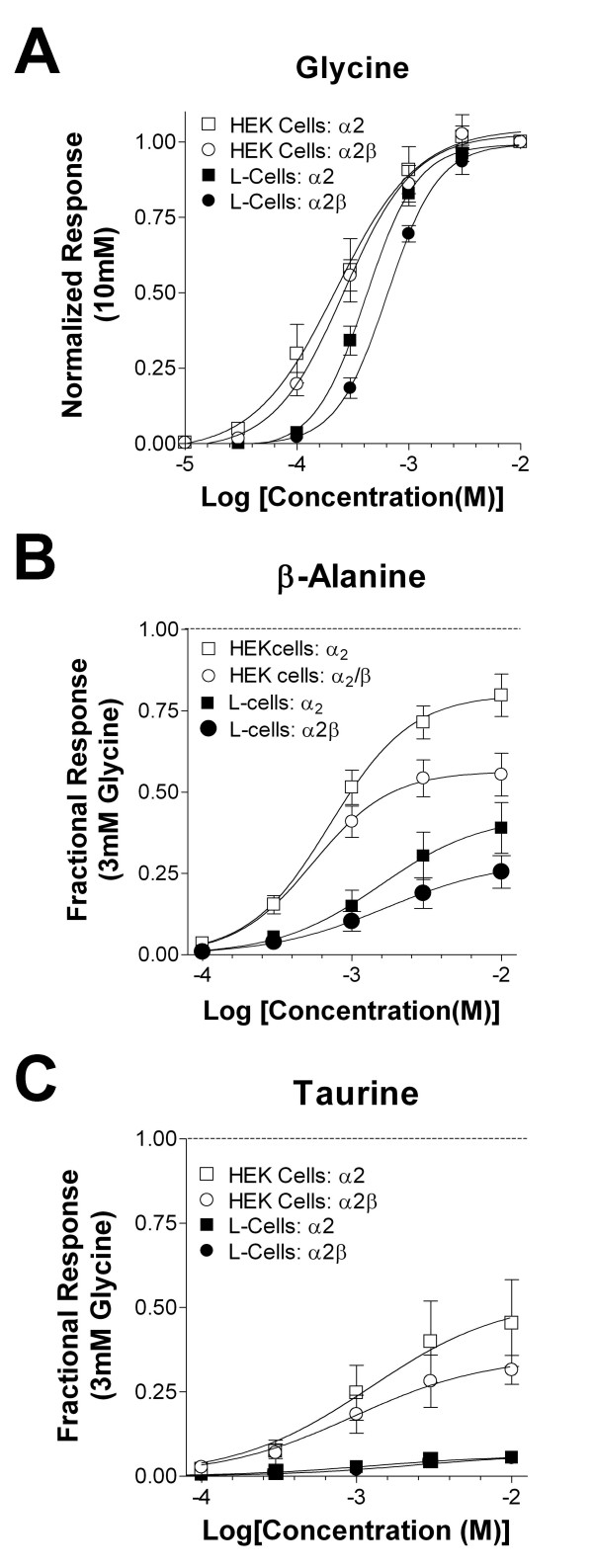
Glycine receptors expressed show expression-system dependent agonist pharmacology. **(A) **Glycine has a reduced potency in L-cells compared to HEK cells. Glycine current responses were plotted versus the log concentration of glycine and normalized to a maximal concentration of glycine (3–10 mM). Data are presented as mean ± SEM; 4 ≤ n ≤ 9 cells for each concentration. Concentration response relationships for GlyRα_2 _(□; EC_50 _= 221 μM) and GlyRα_2_β (○; 269 μM) in HEK cells and in L-cells (GlyRα_2_, ■, 446 μM; GlyRα_2_β, ●; 667 μM) were derived from logistic equation fits to individual cells. **(B) **β-alanine has both reduced apparent affinity and efficacy for most glycine receptor isoforms transiently expressed in L-cells compared to HEK 293 cells. β-alanine apparent potency in HEK 293 cells was 717 μM for GlyRα_2 _(□, n = 6) and 560 μM for GlyRα_2_β (○, n = 7). For L-cells, β-alanine potency for GlyRα_2 _(■, n = 5–8) was 1.61 mM and 1.79 mM for GlyRα_2_β (●, n = 7). Current responses were normalized to a maximal concentration of glycine (10 mM). Note the reduced apparent efficacy of α_2_β receptors compared to the α_2 _homomeric isoforms. **(C) **Taurine has both reduced apparent affinity and efficacy to GlyRs transiently expressed in L-cells compared to HEK 293 cells. Taurine concentration-response relationship in HEK 293 for GlyRα_2 _(□; 442 μM, n = 5–6) and GlyRα_2_β (○; 1.25 mM, n = 3–5). Taurine concentration-response relationship in L-cells yielded GlyRα_2 _(■, n = 4) and GlyRα_2_β (●, n = 7) potencies estimated at ≥ 3 mM. Current responses were plotted versus the log concentration of taurine and normalized to a maximal concentration of glycine.

Given the variation of glycine receptor partial agonist pharmacology in the literature, we specifically sought to identify any role that expression system may play in their pharmacological profiles. First, glycine concentration-response relationships were established for GlyRα_2_, and GluRα_2_/β in HEK-293 cells and in L-cell fibroblasts. Glycine-gated responses for each receptor isoform were elicited in a dose-dependent manner in both cell types (Fig. 1A). The apparent EC_50 _of glycine HEK cells was 221 μM and 269 μM for α_2 _(n = 4–6) and α_2_β (n = 7–8), respectively. GlyR subunits expressed in L-cells displayed a similar pharmacological profile. However, the apparent glycine EC_50 _of both GlyRα_2 _(446 μM, n = 5–7) and GlyRα_2_β (667 μM, n = 4–8) appeared lower than apparent affinities for the same subunits when expressed in L-cells. Two-way ANOVA on the Log (EC_50_) values (Table 1) indicated a significant effect of system (F = 20.01, P < 0.001). However, the presence of the β-subunit did not significantly affect glycine apparent affinity in either system nor was there a significant interaction between system and subunit composition. These results indicate that glycine is less potent for receptors expressed in L cells compared to HEK cells.

**Table 1:** Agonist Pharmacology in HEK and L-cells.

	GlyR α2			GlyR α_2_β		
System	log EC_50_	EC_50 _(mM)	Efficacy^a^	log EC_50_	EC_50 _(mM)	Efficacy
	Glycine					
HEK	-3.67 ± 0.15	0.22	--	-3.55 ± 0.06	0.28	--
L-cells	-3.36 ± 0.05	0.43	--	-3.17 ± 0.04	0.67	--
	β-Alanine					
HEK	-3.12 ± 0.05	0.76	0.80 ± 0.06	-3.24 ± 0.03	0.57	0.55 ± 0.07
L-cells	-2.71 ± 0.08^b^	1.93	0.39 ± 0.08	-2.59 ± 0.13	2.59	0.25 ± 0.05
	Taurine					
HEK	-3.13 ± 0.12	0.74	0.48 ± 0.12	-2.67 ± 0.12	2.20	0.32 ± 0.05
L-cells	≥-3.00 ± 0.11^c^	1.00	0.06 ± 0.01	≥-2.97 ± 0.20^c^	1.10	0.05 ± 0.01

a – efficacy relative to maximal glycine response b – p < 0.0001 for system but not subunit using two-way ANOVA c – estimate due to low efficacy

### Subunit- and system-dependent effects on β-Alanine pharmacology

With the glycine pharmacological profile established, we next examined the pharmacology of the partial agonists, β-alanine and taurine. The efficacy and potency of these β-amino acids were compared to glycine by normalizing the current response at each concentration to a maximal glycine response in that same cell. In HEK cells (Fig. 1B), the average β-alanine EC_50 _values calculated from individual cells were 770 μM (n = 6) and 570 μM (n = 7) for α_2 _and α_2_/β receptors, respectively. In L-cells, β-alanine also elicited currents in a dose-dependent manner. And, like glycine, β-alanine appeared to be less potent in these cells compared to HEK cells. EC_50 _values for α_2 _and α_2_/β receptors were 2.0 mM (n = 8) and 2.9 mM (n = 7), respectively. Two-way ANOVA on LogEC_50 _values from these studies indicate a significant effect of the expression system on β-alanine potency (F = 43.52, P < 0.0001). There was a trend for the presence of the β-subunit to influence potency but this was not significant nor was there any significant interaction between expression system and subunit composition.

β-alanine efficacy was also examined in these same experiments by normalizing the maximal β-alanine response as a fraction of a maximal glycine response. In HEK cells, the α_2 _and α_2_β isoforms had efficacies of 80 ± 6% and 55 ± 7% of the maximal glycine response, respectively. A similar trend was noted in L-cells with the α_2 _and α_2_β isoform with β-alanine efficacies being 39 ± 8% and 25 ± 5% of the maximal glycine response. Two-way ANOVA analysis of these data indicate that both expression system and subunit composition had a significant influence on β-alanine efficacy (F = 27.6, P < 0.0001 and F = 7.9, P < 0.01 respectively). There was no significant interaction between these variables. These data demonstrate that the presence of the β subunit reduced β-alanine efficacy of α_2_-containing receptors and that this efficacy was substantially smaller L-cells compared to HEK cells.

### Subunit- and system-dependent effects on taurine pharmacology

Similar analysis of taurine pharmacology in HEK and L-cells revealed more dramatic effects of system and subunit on this partial agonist (Fig. 1C). In HEK cells, the apparent EC_50 _for taurine was 501 μM for GlyRα_2 _(n = 9) and 2 mM for GlyRα_2_β (n = 7). Because of its remarkably low efficacy in L-cells (see below), we can only provide estimates of taurine potency in this expression system. Regardless, apparent taurine affinity for both GlyRα_2 _and GlyRα_2_β expressed in L cells were ~3 mM for both isoforms (n = 4 and 7, respectively). We did not compare L cell data with that obtained from HEK cells due to the uncertainty surrounding the fits. However, there was no significant difference in apparent taurine potency between the α_2 _and α_2_β receptors expressed in HEK cells (P >> 0.05, t-test).

Taurine efficacy was obviously quite different between the two expression systems. In HEK cells, taurine efficacy was 48 ± 12% of glycine for GlyRα_2 _and 32 ± 4% of glycine for the GlyRα_2_β isoform. Efficacy for these same receptors was reduced to approximately 6 ± 1% and 5 ± 0.7% of glycine when they were expressed in L-cells in these particular studies. The system difference was significant with two-way ANOVA (F = 17.4, P < 0.001) with no substantial effects of subunit composition or interactions between these variables.

### Expression level and system-dependent pharmacology

**Figure 2:**
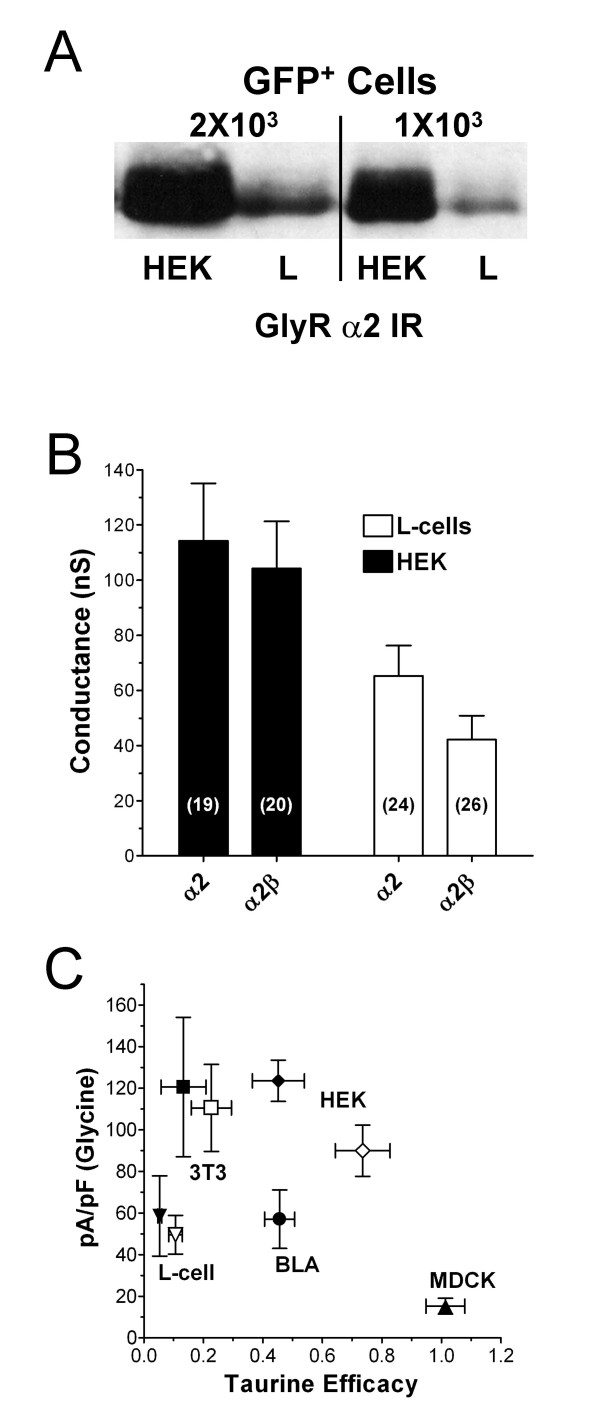
Relative expression levels does not influence taurine efficacy. **(A) **HEK and L-cells were co-transfected with the GlyRα_2 _subunit and GFP. Relative expression levels of α_2_-protein were examined using western blot analysis of total lysate from equal numbers of GFP^+ ^HEK and L-cells. α_2 _protein was 4- to 5-fold greater in GFP^+ ^HEK cells than in GFP^+ ^L-cells. **(B) **Maximal glycine conductance across all experiments was significantly lower in L-cells compared to HEK cells, although only by about 2-fold. This may indicate that a significant amount of α_2 _protein in HEK cells (A) is present in a non-functional form or not associated with the plasma membrane. **(C) **Glycine current density vs. taurine efficacy in different cell lines expressing α_2 _(open symbols), α_2_β glycine receptors (closed symbols), and in isolated neurons from the adult rat basolateral amygdala. The correlation coefficient between glycine current density and taurine efficacy was 0.14 and was not significantly greater than zero (P >> 0.05). There was also no correlation (R^2 ^= 0.01 to 0.3) between glycine current density and taurine efficacy when comparing individual cells *within *each of these systems.

The preceding results suggest that there may be a complex interaction between subunit composition and the expression system in which the receptor is produced. Specifically, the system-dependent agonist pharmacology could be related to differences in the relative expression levels between various systems. Expression level has clearly been demonstrated to influence agonist pharmacology for G protein-coupled receptors (e.g. [13]), where the levels of G-protein bound to receptor and thus the relative levels of high affinity receptor can vary from system to system. However, the influence of expression level on ligand-gated channel function has not been extensively explored (see Discussion). Unfortunately, it is problematic to compare expression levels between HEK and L-cells since the relative efficiency of transfection varied widely between these systems. Indeed, liposome-mediated transfection is remarkably efficient in HEK 293 cells (70–90% of cells based on GFP fluorescence) but only marginally effective in L-cells (10–20% of cells, not shown). To get around these differences in transfection efficiency, we examined the relative expression level of GlyRα_2 _protein using western analysis of total lysate derived from the same number of GFP^+ ^HEK 293 or L cells from transfected cultures (Fig. 2A). For this experiment, cells were harvested under native conditions, GFP^+ ^cells were counted, and volumes of lysate corresponding to equivalent numbers of GFP^+ ^cells was loaded onto the gel. Western blots from two separate experiments demonstrate that transfected HEK 293 cells expressed 4- to 5-fold more GlyRα_2 _protein than transfected L-cells. The mean optical density from the two experiments was 83 ± 2 units for HEK cells and 17 ± 2 units for L-cells.

Maximal conductance is an independent measure of functional expression and was also larger for both α_2 _and α_2_β receptors expressed in HEK cells compared to receptors expressed in L cells (Fig. 2B). Across all experiments where maximal glycine concentrations were assayed, the conductance of α_2 _receptors expressed in L-cells was 65 ± 11 nS and was 114 ± 21 nS in HEK cells. Similarly, L-cells expressed α_2_β receptors at 42 ± 9 nS while HEK cells expressed this isoform at 104 ± 17 nS. Two-way ANOVA using subunit and system as variables revealed a significant effect of system (F = 15.4, P < 0.001) but not subunit, nor was there a significant interaction between variables. Results from both westerns and functional experiments therefore indicate that relative expression levels of glycine receptor were different between HEK and L-cells.

To further explore the interaction between expression level and partial agonist efficacy, both current density and taurine efficacy were compared for α_2 _and α_2_β glycine receptors in a number of different heterologous systems, as well as for native receptors expressed in rat lateral/basolateral amygdala. In addition to HEK and L-cells, the heterologous systems included mouse 3T3 fibroblasts and MDCK kidney cells. α_2_β receptors expressed in mouse 3T3 fibroblasts had twice the current density (121 ± 34 pA/pF) of the mouse L-cells (59 ± 19 pA/pF) but had a similar taurine efficacy (13 ± 8% of glycine in 3T3 cells versus 8 ± 1% in L-cells). Similarly, α_2_β receptors expressed in HEK293 cells had a current density similar to GlyRs expressed in 3T3 fibroblasts (115 ± 11 pA/pF) but had a taurine efficacy compared to glycine of 48 ± 3%. This efficacy was similar to glycine receptors expressed by acutely isolated adult rat basolateral amygdala neurons (46 ± 5% of glycine) although the current density in this native system was only 57 ± 14 pA/pF. Note that the channels expressed by these neurons are composed primarily of α_2_+β subunits [4]. Canine kidney MDCK cells expressed the lowest α_2_β current density (15 ± 5 pA/pF); yet the channels expressed by this system had the highest taurine efficacy of any cell tested (101+6%). For α_2 _GlyRs, the rank order of glycine receptor density was 3T3 (111 ± 21 pA/pF)> HEK cell (90 ± 11 pA/pF)> L-cell (50 ± 9 pA/pF); while the rank order of taurine efficacy for these same receptors was HEK (74 ± 9%)> 3T3 (23+7%)> L-cells (11 ± 2%). Across all subunit combinations and systems, there was no significant correlation (R^2 ^= 0.14, P >> 0.05) between taurine efficacy and glycine current density (Fig. 5C). Indeed, no correlation between expression level and taurine efficacy was evident within any given population of cells whether the receptors were expressed in native or heterologous systems. For example, the correlation coefficients for α_2_β receptors between taurine efficacy and glycine current density in individual systems were 0.11, 0.13, 0.19, 0.05, and 0.31 for HEK, L-cells, 3T3 cells, MDCK cells, and amygdala neurons, respectively (P >> 0.05). Thus, while there is clearly a difference in expression level between both the systems as well as between individual cells in a given system, this particular characteristic cannot account for the apparent taurine efficacy.

### The agonist-binding site is not affected by expression system

**Figure 4:**
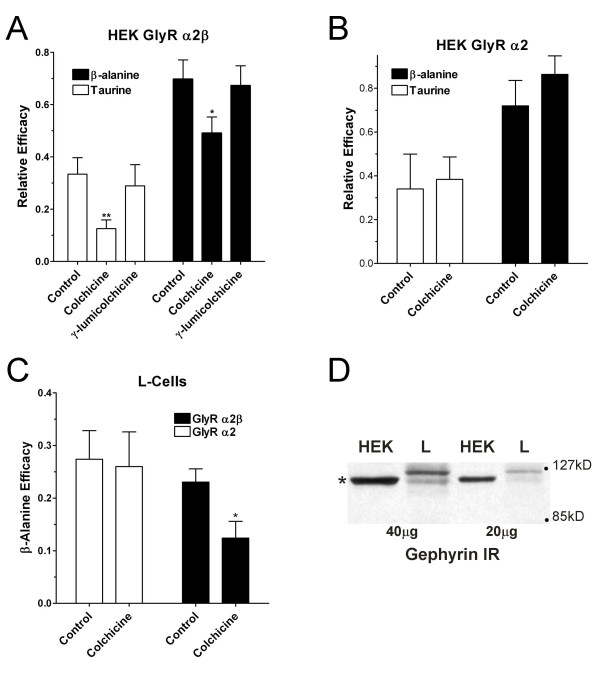
The association of glycine receptors with the tubulin-cytoskeleton may influence partial agonist efficacy. **(A) **Tubulin depolymerization with colchicine decreased both taurine and β-alanine efficacy of α_2_β glycine receptors expressed in HEK 293 cells. Cells were treated with 100 μM colchicine or γ-lumicolchicine at 37°C for 30 minutes. The graph shows the partial agonist efficacy as a fraction of the maximal glycine response. For taurine (□), colchicine treatment reduced apparent efficacy from 33 ± 6% in control cells (n = 8) to 13 ± 3% in treated cells (n = 10). γ-lumicolchicine, an inactive analogue of colchicine, had no effect on taurine efficacy (29 ± 9%, n = 5, ** – P < 0.01 from ANOVA). For β-alanine (■), efficacy was reduced from 70 ± 7% in vehicle-treated cells (n = 8) or 67 ± 8% in γ-lumicolchicine-treated cells (n = 5) to 49 ± 6% in cholchicine-treated cells (n = 10, * – P < 0.05, ANOVA). **(B) **Colchicine treatment does not influence partial agonist efficacy of the GlyRα_2 _homomeric channels. Taurine (□) efficacy was 34 ± 15% in control GlyRα_2 _cells (n = 4) and was 38 ± 10% in colchicine-treated cells (n = 5, P >> 0.05 t-test). Similarly, β-alanine efficacy was 72 ± 12% and 86 ± 8% in the same control and treated cells, respectively (P >> 0.05, t-test). **(C) **Colchicine treatment decreases β-alanine efficacy in L-cells expressing GlyRα_2_β heteromeric channels (■), but not those expressing GlyRα_2 _homomeric channels (□). For the GlyRα_2_β channels, colchicine treatment significantly reduced efficacy from 23 ± 2% (n = 7) to 12 ± 3% (n = 3, P < 0.05 t-test). **(D) **Gephyrin-like immunoreactivity was detected in both cells lines using 20 and 40 μg of whole cell lysate. * – denotes expected gephyrin mobility (approx. 100 kD).

There are a variety of possible mechanisms to account for the disparities in partial agonist pharmacology between two expression systems. One way to address this is to examine competitive antagonist binding properties in HEK and L-cells. We therefore examined the potency of the glycine receptor competitive antagonist strychnine in both systems. Following a 30 second pretreatment with the antagonist [2], we co-applied strychnine and an EC_50 _concentration of glycine. The strychnine K_B _was estimated for HEK and L-cells expressing either the GlyRα_2 _or GlyRα_2_+β subunits using the Cheng-Prusoff relationship (see Methods). This relationship takes into account the divergent Hill-slope and potencies for glycine found in these two expression systems. Receptors composed of the GlyRα_2 _subunit (Fig. 4A) had very similar K_B _values when expressed in either HEK (K_B _= 49 ± 8 nM, n = 11) or L-cells (K_B _= 38 ± 7 nM, n = 16; Fig. 4C). The same was true for cells expressing the GlyRα_2_β subunits where strychnine apparent affinity was 32 ± 7 nM (n = 10) in HEK cells and 38 ± 8 nM (n = 10) in L-cells. Two-way ANOVA did not reveal any significant effect of either system or subunit composition. Since strychnine is a competitive antagonist and site-directed mutation studies suggests that strychnine and glycine interact with overlapping regions of the receptor [14-16], our results strongly suggest that functional strychnine affinity, and hence the general structure of the agonist binding pocket, was not substantially influence by expression system.

### Cytoskeletal components influence glycine receptor pharmacology

A third possible mechanism for reduced efficacy in L-cells compared to HEK cells or neurons could be related to intracellular factors that influence channel gating [17]. This hypothesis was examined by disrupting the cytoskeletal protein tubulin, which has been shown to be important for glycine receptor localization [18]. Direct application of 100 μM colchicine did not elicit any membrane currents. Furthermore, acute application of 100 μM colchicine and an EC_50 _concentration of glycine (300 μM) did not significantly affect glycine-gated currents themselves. Glycine currents were 17.3 ± 2.3 pA/pF while glycine+colchicine currents were 16.7 ± 2.2 pA/pF (p > 0.5, paired two-tail t-test, n = 7).

The relative efficacy of β-alanine and taurine was examined in HEK cells expressing α_2_β subunits following 30 min incubation with 100 μM colchicine at 37°C, enough time to allow irreversible tubulin disruption [19]. As an additional control, γ-lumicolchicine, an inactive analog of colchicine [20], was also used to treat α_2_β-expressing HEK cells (Fig 4A). These brief treatments had no obvious effect on the survival of untransfected cells. There was a trend for colchicine treatment to reduce the overall current density at 300 μM glycine, 56.7 ± 9.1 pA/pF in control cells (n = 8), 41.2 ± 2.3 pA/pF in colchicine-treated cells (n = 10), and 50.1 ± 9.9 pA/pF (n = 6) in γ-lumicolchicine-treated cells; however, this was not significant (p > 0.05, ANOVA) and was probably not related to any direct action of colchicine given that the glycine current density was also slightly reduced in α_2_β-expressing cells exposed to γ-lumicolchicine compared to controls. However, the efficacy of both taurine (p < 0.01, One-way ANOVA) and β-alanine (p < 0.05, ANOVA) were significantly decreased by colchicine but not γ-lumicolchicine treatment. Taurine efficacy was 33 ± 6% of glycine in controls, 13 ± 3% following colchicine, and 28 ± 3% following γ-lumicolchicine. Similarly, β-alanine efficacy was 70 ± 7% of glycine in controls, 49 ± 6% following colchicine, and 72 ± 7% following γ-lumicolchicine. Similar treatment of α_2_-expressing HEK cells with colchicine (Fig. 4B) did not reveal any significant effect on glycine current density (54 ± 14 pA/pF in controls, 60 ± 15 pA/pF in treated), on taurine efficacy (34 ± 16% in controls vs. 38 ± 10% in treated), or on β-alanine efficacy (71 ± 12% in controls vs. 86 ± 8% in treated). Cholchicine treatment also significantly reduced β-alanine efficacy in L-cells expressing GlyRα_2_β (23 ± 2% in controls vs. 12 ± 3% in treated, P < 0.05, t-test) but not in GlyRα_2_-expressing L-cells (Fig. 4C). We did not attempt to examine taurine in L-cells treated with colchicine given the exceptionally low efficacy of receptors expressed in this cell line.

Because the glycine receptor- and tubulin-binding protein gephyrin provides an obvious link between the receptor and the tubulin cytoskeleton, we used western analysis of HEK and L-cell lysates with a gephyrin monoclonal antibody specific for the C-terminus. These experiments revealed that gephyrin-like immunoreactivity was expressed in both expression systems (Fig. 4D). Notably, a ca. 100 kD band dominated the HEK cell gephyrin immunoreactivity, while multiple bands of varying intensity could be seen in lysate from L-cells. When taken with our colchicine data, differences in glycine receptor pharmacology between α_2_β receptors expressed in HEK and L-cells may be partially due to distinct, system-dependent interactions with distinct isoforms of the cytoskeletal protein gephyrin.

## Discussion

We have expressed several the 'embryonic' (α_2 _homomeric) and 'forebrain' (α_2_β heteromeric) isoforms in two distinct expression systems to understand the influence of endogenous and exogenous factors on receptor partial agonist pharmacology. Although the pharmacology of the 'embryonic' GlyRα_2 _isoform and the 'adult spinal' isoform (GlyRα_1_β) have been explored more frequently in the literature, the pharmacology of GlyRα_2_β receptors has remained largely unexplored. Despite this, there is strong evidence that the adult 'forebrain' isoforms, specifically in the rat basolateral amygdala, is indeed α_2_β [4]. The current study indicates a general trend for decreased apparent affinity and reduced relative efficacy of agonists when receptors consist of the α_2_β subunits compared to their homomeric α_2 _counter parts.

We were particularly surprised to find that receptors expressed in different expression systems possessed markedly different partial agonist efficacies. The remainder of our study focused on identifying extrinsic factors that influence difference in ligand-gated receptor pharmacology in distinct expression systems. While differences in efficiency of cDNA expression/transfection between systems could explain such differences, the expression levels of GlyRα_2_β receptors measured by current density was not correlated with taurine efficacy across several different cell types or within any given system. Importantly, the efficacy of β-alanine and taurine in HEK cells agree with previous findings where cells expressing GlyRα_2 _show almost full efficacy for taurine and β-alanine [21]. Similarly, distinct ligand binding characteristics of receptors expressed in different expression systems seemed to be another possible mechanism governing agonist efficacy or potency. For the glycine receptor, the binding site for the competitive antagonist strychnine is believed to be adjacent to the agonist-binding site, sterically hindering agonist binding. A gross alteration in the agonist binding pocket, particularly one that hindered agonist binding, would most likely affect strychnine binding as well. To examine this, strychnine K_B _was calculated for GlyRα_2 _and GlyRα_2_β isoforms expressed in both HEK and L-cells. In order to decrease the error in estimating K_B_, a derivation of the Cheng-Prusoff equation was used that takes in account variations in the slopes of the inhibition curves [22]. Differences in K_B _were negligible between expression systems, indicating the strychnine binding site, and presumably the agonist binding site, was altogether similar in these different systems. Differences in pharmacology between systems therefore cannot be explained by substantial alterations in the agonist/competitive antagonist binding pocket.

Receptor gating is another mechanism by which receptor function may be altered. Cytoskeletal elements have been shown to play a crucial role in neurotransmitter receptor clustering [17] and may have a role in receptor function as well. For example, cytoskeletal stabilization has been shown to reduce Ca^++^-dependent inactivation of Ca^++ ^channels in snail ganglia [23]; and, actin has been shown to modulate several different types of membrane ion channel [24-26]. Cytoskeletal depolymerization has also been found to inhibit the function of GABA_A _receptors, which share significant sequence homology and functional characteristics with strychnine-sensitive glycine receptors [27]. And the tubulin-gephyrin-glycine receptor interaction is critical for establishing functional glycinergic synapses [28]. The current study suggests that cytoskeletal elements may play a functional role in α_2_β glycine receptor pharmacology as well. β-containing glycine receptors are intimately associated with the tubulin-associated protein gephyrin [29] via the gephyrin binding site that lies within the intracellular domain of this subunit [30]. In our studies, the efficacy of both taurine and β-alanine were reduced in cells expressing GlyRα_2_β subunits. This despite the finding that gephyrin-like immunoreactivity in L-cells was apparently distinct from that in HEK cells, suggesting that distinct cytoskeletal components in these systems may have profound influence over GlyR α_2_β pharmacology. This is further supported by suggestions that gephyrin may exist in multiple, tissue-specific isoforms with potentially distinct functional roles [31-33]. It should be noted however that colchicine treatment of α_2_β-expressing HEK cells did not suppress partial agonist efficacy to a level that approached that found in L-cells or 3T3-fibroblasts. Given that colchicine had no perceptible effect of α_2_-homomeric channels expressed in HEK cells, our results suggest that additional system-dependent factors may have a more pronounced influence on the partial agonist pharmacology of strychnine-sensitive glycine receptors.

## Conclusions

It is of particular interest that the β subunit appears to play a functional role in the pharmacology of α_2_-containing receptors regardless of expression system. For example, the beta subunit decreased the apparent efficacy of the partial agonists. Since the β-subunit itself does not appreciably interact with the competitive antagonist strychnine [34], these results are at least consistent with some allosteric interaction between the β-subunit and the agonist binding site present the α subunit. This may indicate that α_2_β glycine receptors in the forebrain may be distinguishable from other receptor isoforms given the appropriate pharmacologic agent. Although cytoskeletal components potentially play some role for these 'forebrain' receptors, there appear to be other 'extrinsic' factors governing expression system-dependent effects on agonist pharmacology. Since it is conceivable that such factors may be differentially distributed between different forebrain regions, the large apparent differences between glycine receptor pharmacology reported by various studies may not necessarily depend upon differential expression of glycine receptor subunits per se. At the very least, our findings suggest that great care should be taken when utilizing different expression systems to develop screens for novel pharmacophores acting on this receptor.

## Methods

### Cell culture and transfection

HEK 293 (TSA 201; gift from Michael J. Davis, Dept. Med. Physiol., Texas A&M Univ. Health Science Center, College Station, USA), mouse L-cells (NCTC-929; American Type Culture Collection), NIH/3T3 fibroblasts (ATCC), and MDCK cells (gift from Alan Parrish, Dept. Med. Pharmacol. & Toxicol., Texas A&M Univ. Health Sci. Center) were grown in Dulbecco's modified Eagle's medium (DMEM, SIGMA) with 10% fetal bovine serum (HyClone Laboratories, Logan, UT, USA) and 1X penicillin/streptomycin (Life Technologies) on Thermanox cover slips in 35 mm culture dishes. Cells were transfected during log-phase growth (30–50% confluent) using the Superfect reagent (Qiagen, Valencia, CA, USA), according to manufacturers instructions. Rat glycine receptor α_2 _and β subunits were cloned previously [4]. Briefly, a total of 2–3 μg of plasmid constructs was added to 100 μL serum-free media along with 10 μL Superfect reagent, vortexed for several seconds, and incubated for 10 minutes to allow DNA/liposome formation. Standard media (600 μL) was added and this mixture applied the cells and incubated for 2–3 hours at 37°C. The cells were subsequently washed twice with phosphate-buffered saline, fresh media was applied and cells were then incubated for 24–48 hours prior to recording. Cells were co-transfected with green fluorescent protein (pEGFP-C1, Clontech, Pal Alto, CA, USA) to identify transfected cells before recording. Mass ratios of 1:1:5 were used in the GFP:α_2_:β transfections. An equal mass of the cloning vector pCI (Promega Corp) replaced the β subunit when examining α_2 _homomeric channels.

### Electrophysiology

Whole cell recordings were performed at room temperature using standard patch-clamp techniques and the axopatch-1D amplifier (Axon Instruments, Inc., Foster City CA, USA) in the voltage clamp mode. Gigaohm seals were formed using patch pipettes made from borosilicate glass (World Precision Instruments, Sarasota FL, USA). For most experiments, the internal solution contained (in mM): CsCl 100, EGTA 11, HEPES 10, CaCl_2 _1, Mg-ATP 4, pH 7.2 with methane sulfonic acid; adjusted to 290–295 mmol kg^-1 ^with sucrose. Whole cell capacitance and series resistance was manually compensated after opening the cell. Cells were continuously bath perfused with a HEPES-buffered saline (in mM): NaCl 150, Glucose 10, HEPES 10, KCl 2.5, CaCl_2 _2.5 MgCl_2 _1.0, pH 7.4, 305–320 mmol kg^-1 ^Data will be analyzed off-line using pClamp software (Axon). Numerical analysis was performed using commercially available software. Independent student's t-test and two-way ANOVA were used for comparisons where appropriate; and statistical significance was based on p < 0.05. Concentration-response curves were generated from fits of data to a standard logistic equation as previously described (McCool & Botting, 2000). To derive K_B _from functional IC_50 _and EC_50 _data, the Cheng-Prusoff equation was used:



where *S *= hillslope of agonist curve, *A *= concentration of agonist, EC_50 _= half-maximal agonist concentration, and IC_50 _= half-maximal antagonist concentration.

### Drugs

Stocks of glycine, taurine, β-alanine, strychnine (Tocris) and colchicine (SIGMA) were prepared fresh each day. Agonists and antagonists were applied for 4–10 sec from an array of eight HPLC-grade capillary tubes (150 μm i.d.; Hewlett Packard Analytical Direct) placed within 100 μm of the cell of interest.

### Western analysis

Cells were cultured, transfected as stated above except in 10 cm Petri dishes, and harvested by scrapping with 500 μL of lysis buffer (10 mM Tris pH 7.5, 1% SDS). Proteins were quantified using the Bradford assay and loaded onto 8–10% SDS-polyacrylamide gels, separated, and transferred to a nitrocellulose membrane (Hybond C, Amersham). The membrane was blocked overnight TBS (200 mM NaCl, 10 mM Tris pH 7.5) containing 0.2% Tween-20 and 10% low-fat dry milk. Blots were then incubated in TBS/0.1% Tween-20 containing primary GlyR antibody (monoclonal GlyR4a antibody, 1:200, Alexis Biochemicals) and monoclonal anti-gephyrin antibody (1:2000, Transduction Laboratories) for two hours at room temperature. After several washes, the HRP-coupled rabbit anti-mouse secondary antibody (SIGMA) was added for one hour (1:2000). The detection was performed by the ECL method (Amersham).

## List of abbreviations

GlyR – glycine receptor; BLA – lateral/basolateral amygdala; HEK – human embryonic kidney cells; MDCK – Manin-Darby canine kidney cells

## Author's contributions

JF carried out the electrophysiological recordings and participated in the western analysis. BM conceived of the study, participated in its design and coordination, performed the western analysis and some electrophysiology experiments, and drafted the manuscript. All authors read and approved the final manuscript.

**Figure 3:**
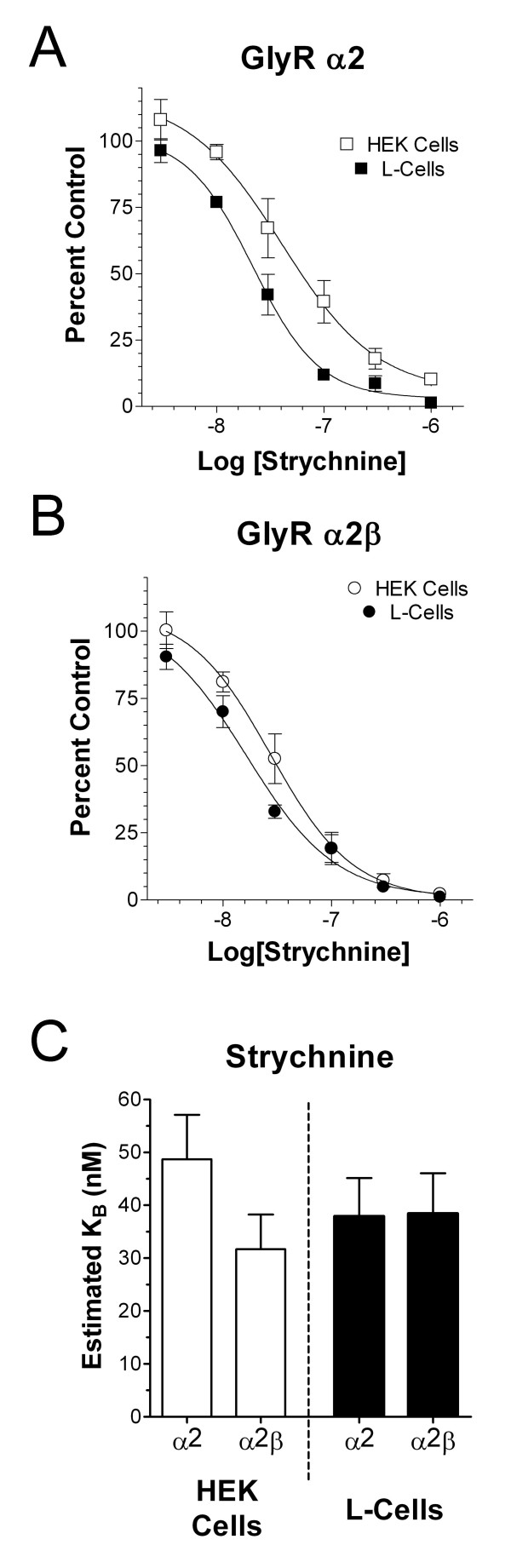
Glycine receptors expressed in different expression systems have similar 'functional' strychnine binding. Cells were pre-treated for 30 seconds with strychnine alone then exposed to a strychnine admixture with an EC_50 _concentration of glycine. **(A) **Strychnine-mediated inhibition of glycine-gated currents of GlyRα_2 _homomers expressed in HEK 293 (□; IC_50 _= 78.2 ± 13.5 nM) and L-cells (■; 33.1 ± 6.3 nM). **(B) **Strychnine-mediated inhibition of glycine-gated currents of GlyRα_2_β heteromers expressed in HEK 293 (○; 37.9 ± 7.9 nM) and L-cells (●; 23.2 ± 4.6 nM). **(C) **Functional K_B _values were calculated from IC_50 _values for individual cells using the Cheng-Prusoff relationship and glycine affinity/Hillslope data represented in Figure 1. Average K_B _values are shown for each subunit combination in the two expression systems. There was no significant effect of subunit compositions or expression system.
